# Evaluation of the Surface Irregularities of the Cross-Section of the Wood after CO_2_ Laser Cutting

**DOI:** 10.3390/ma16227175

**Published:** 2023-11-15

**Authors:** Lukáš Adamčík, Rastislav Igaz, Lukáš Štefančin, Ivan Kubovský, Richard Kminiak

**Affiliations:** Faculty of Wood Sciences and Technology, Technical University in Zvolen, T.G. Masaryka 24, 96001 Zvolen, Slovakia; xadamcikl@tuzvo.sk (L.A.); igaz@tuzvo.sk (R.I.); xstefancin@tuzvo.sk (L.Š.); kubovsky@tuzvo.sk (I.K.)

**Keywords:** CO_2_ laser cutting, primary profile, surface irregularities, cross-section, digital microscope

## Abstract

The present paper deals with the analysis of cross-section surface irregularities after CO_2_ laser cutting. The surface irregularities of beech (*Fagus sylvatica* L.), oak (*Quercus petraea*), and spruce (*Picea abies* L.) wood were quantified by primary profile parameters using a digital microscope. The arithmetic mean height (Pa), used as the basic parameter, was supplemented by amplitude parameters (Pv, Pp, Pz) and the Psm parameter, through which the shape of the irregularity was specified in more detail. A statistically significant change was demonstrated when changing the values of the feed speed and the power of the CO_2_ laser. The results of this article confirm that the surface irregularities increased with an increasing laser power and decreasing feed rate. The scanned topographic images also provide a more detailed explanation of the measured P-parameters and point out the risks associated with the evaluation of the cross-section with the primary profile.

## 1. Introduction

Laser cutting emerged as a commercial processing technique in the early 1970s. Since its inception, it has pioneered advancements in industrial processing and has become a dominant force in the commercial market [1].

Currently, laser technology is used in the automotive industry, aviation, shipbuilding, arms industry, energy, and many other industries, mainly for welding, melting, engraving, or cutting [2]. Wood cutting itself was one of the first commercial applications of lasers [3,4,5].

In contrast to traditional cutting methodologies, laser cutting presents notable advantages encompassing cutting accuracy, flexibility, automation, and efficiency, in conjunction with a nearly waste-free, wear-free, and force-free process. Significantly, laser cutting demonstrates enhanced environmental amicability by facilitating a markedly quiet operation, thereby mitigating noise pollution. Additionally, the intersection of environmental and economic considerations is noteworthy. The operational expenditure associated with laser cutting is comparatively lower when juxtaposed with conventional cutting methodologies, thereby presenting a cost-effective and environmentally responsible alternative for industrial applications [6].

Laser beam cutting has become another alternative method of wood cutting along with the water jet [7]. Generally, laser beam capacity to cut wood is linked to the absorption of light, the conversion of this light energy into heat, the distribution and rate of heat transfer within the material, and the rate of vaporization in the irradiated area [8]. In the field of furniture production, laser cutting has become popular, for example, for surface engraving [9,10]. The main advantages of laser cutting are a highly precise cut, the possibility to start and finish the cutting trajectory at any point in the board and a narrow cutting gap compared to rotary tools [11,12,13].

Despite the advancements in CO_2_ laser cutting technology, the post-cutting surface quality of timber remains a concern that warrants a thorough investigation. Surface roughness or waviness is a critical parameter as it affects not only the aesthetic appeal but also the functional properties of the final product, such as bonding strength for gluing, coating adherence, and the overall finish quality [1,14]. While its application has been extensively studied in metals and polymers [15], the intricacies of laser cutting wood, as a natural and anisotropic material, presents a unique set of challenges when subjected to laser cutting. Factors such as moisture content, grain direction, and wood species can greatly influence the cutting process, resulting in varying outcomes in terms of cut quality, efficiency, and material behavior. As the demand for refined wood products grows, understanding these interactions is paramount to optimizing laser cutting parameters and ensuring product quality [16,17,18,19].

In a recent study by Guo et al. [20], the CO_2_ laser cutting of pine wood was explored, revealing key insights into the kerf surface morphology, processing-induced defects, and the combined mechanism of the laser beam and gas jet, emphasizing the potential of CO_2_ lasers in wood cutting and its implications for efficiency and yield. In a study by Castañeda et al. [21], on the laser cutting of pine wood, it was found that the quality and efficiency of the cut were significantly influenced by the moisture content, cut direction relative to the wood’s grain, traverse speed, and laser power. Eltawahni et al. [22,23] explored the optimal parameters for the laser cutting of board materials such as MDF—medium density fiberboard and plywood, emphasizing the intricate interplay between various factors to achieve the highest quality cuts, providing a foundational reference for this research on the timber surface roughness post-laser cutting. In their investigation of bamboo laser cutting, Xu et al. [24] found that certain settings, including a 48 W laser output power and a cutting speed of 30 m.min^−1^, yielded an optimal kerf quality, setting a precedent for assessing surface outcomes in laser-cut timber. Various studies focused on the impact of CO_2_ laser processing parameters on the surface roughness. Rezaei et al. [16] investigated the impact of parameters such as cutting speed, focal-point positions, and gas pressure on the surface roughness of laser beech wood. Results show that the cutting speed and focal-point position has little to no effect on surface roughness while the increase in gas pressure lowered the average surface roughness.

Since laser cutting is often a curvilinear process of cutting wood, in practice, it is impossible to avoid the surface of the cross-section in the resulting surface. The mutual interaction of the laser with the cellular elements present in the cross-section causes the formation of characteristic irregularities, the research on which is also dealt with by other authors. The very evaluation of these irregularities becomes questionable from the point of view of the combined effects of anatomy and cutting. In a radial or tangential cut, unevenness is evaluated through the roughness profile [16,25,26,27,28]. However, in the case of a cross-section, it becomes insufficient, since through the most used filter value λc = 2.5 mm, there is a significant attenuation of the amplitude for a wavelength of irregularities greater than 2.5 mm. However, if the height irregularities between the less dense earlywood and the more dense latewood after machining are defined as anatomical waviness [29], it is necessary to deal with the irregularities with a wavelength higher than 2.5 mm. The most common width of annual rings, within which the difference in earlywood and latewood will show, is in our climate zone in the range of 2–5 mm. For this reason, the wavelength subject to filtering will also be in the range of 2–5 mm. By changing the filter λc to a value of 8 mm, the transmission band will be widened, but the differences between earlywood and latewood will be defined as roughness. If the combined roughness after CO_2_ laser cutting on the cross-section was measured via waviness, there would be a very significant attenuation of short-wavelength roughness (below 2.5 mm). In such a case, height differences between earlywood and latewood would be defined as anatomical waviness. However, the measurement would miss surface irregularities created by transversely cut cellular elements that behave like dales on the surface. In the same way, irregularities created by melts on the surface would also be suppressed from the measurement of W-parameters. The solution to the aforementioned phenomena could be the measurement of the primary profile. The latter is created using the λs filter, which at a value of 8 μm, only attenuates irregularities with very small wavelengths (below 8 μm). The evaluated profile is thus only minimally affected by the filtration process. Both phenomena will be included in the calculation of P-parameters—irregularities due to laser cutting and irregularities due to anatomical structure. The primary profile could thus become the answer to the question of the difficult evaluation of the quality of the cross-section, as long as the quality is precisely defined by the irregularities.

Even though the study of laser action was dealt with by several authors [16,29,30], this paper aims to find out in more detail the impact of the feed speed and power of the CO_2_ laser on the quality of the surface, which the authors expressed through the primary profile. In the work by Kúdela et al. [31], the radial surface engraved at different laser power values was evaluated. The power output used in the process was lower compared to laser cutting. As a result, the effect of higher outputs and higher energy delivered to area has still unknown results for development of surface irregularities on spruce, oak, and beech wood. The work by Kúdela et al. [30] also evaluated the surface through roughness parameters. Rezaei et al. [16] also dealt with laser cutting. The authors expressed the quality of the surface with the parameter Ra, while the power of the CO_2_ laser was significantly higher (3500 W). Under these cutting conditions, the authors did not detect a statistically significant influence of the laser feed rate. The tangential surface was laser cut. In the work by Gurau et al. [29], the cross-section was evaluated using roughness and waviness parameters. Thus, the idea appeared to divide the irregularities created by the laser into the areas of micro-irregularity and macro-irregularity. The collective of authors investigated the effect of laser cutting parameters on the quality of the cross-section, but with a higher performance and higher feed speed. However, it is not clear from the mentioned works how a smaller feed speed can affect the cross-sectional area using CO_2_ lasers, the power of which is significantly lower (commercial ones, which are very often used in production practice). However, the determination of the methodology for evaluating the quality of the transverse cut becomes necessary, since the transverse cut is an integral part of the transverse or curvilinear cutting of wood. Only a few authors dealt with the definition of the surface quality through the primary profile, but not in connection with laser cutting. It is the methodology of quality assessment through the primary profile that could become the answer to the question of how to comprehensively evaluate the entire range of irregularities of different sizes, while individual irregularities have a different cause (as a result of laser action, and as a result of wood anatomy).

The aim of this paper is to comprehensively analyze the cross-section irregularities that arose when cutting spruce, oak, and beech wood with a CO_2_ laser and to determine in detail the impact of changing the laser power and feed speed. The aim of this paper comprehends the following partial tasks:

- Design and verification of a methodology for measuring the irregularities of the cross-section through the parameters of the primary profile, which would contain two types of irregularities: the first, caused by the anatomical microscopic structure of the cross-section and the second, caused by the changing values of the power and the feed speed of the CO_2_ laser.
- Determining the appropriateness of using the optical method of measuring irregularities on a cross-section and pointing out the risks associated with assessing the quality of a cross-section through P-parameters.
- Identification of dominant irregularities on the cross-section through parameters Pa, Pv, Pp, Pz, and Psm.
- Explanation of the origin of individual irregularities in connection with the anatomical structure of wood using microscopic analysis.

## 2. Materials and Methods

### 2.1. Preparation of Sanded Beech Wood Samples

Three samples with dimensions of 5 × 70 × 500 mm (thickness × width × length) were made of oak wood (*Quercus petraea*), beech wood (*Fagus sylvatica* L.), and spruce wood (*Picea abies* L.) with a radial surface. The mentioned wood species were chosen as representative species for coniferous, ring-porous, and diffuse-porous wood species. The thickness of the sample of 5 mm was chosen as the most optimal value for cutting the grown wood due to the performance of the used CO_2_ laser, where it is possible to cut the material with one pass of the laser beam. The value of the width and length of the samples was chosen considering the optimal yield from oak, spruce, and beech boards as input material. The samples were dried to an equilibrium wood moisture content of 8–10%. The chosen humidity of the samples follows the most frequently achieved equilibrium humidity of the wood under production and operating conditions (the interior of the buildings in which the wooden products will be placed). Before laser cutting, the boards were milled on a jointer and planer. The kerfs cut in the direction perpendicular to the fibers with a length of 50 mm were subsequently cut with a CO_2_ laser CM-1309 (Shenzhen Reliable Laser Tech, Shenzhen, China) with a maximum power of 135 W. The laser power was 50%, 75%, and 100%. A laser power of 100% was chosen as the maximum limit of the CO_2_ laser power, i.e., the highest amount of energy delivered per unit area. At this limit, the largest burn-off of the wood on the cross-section and thus the highest values of irregularities are also expected. The minimum value for cutting through growing wood, as recommended by the manufacturer, is 50% of the laser power value. The 75% laser power value was chosen as an average, optimal value. The selected feed speeds were 5 mm·s^−1^, 10 mm·s^−1^, and 20 mm·s^−1^. The highest value of 20 mm·s^−1^ was chosen as the limit value for cutting the growing wood with one pass of the laser beam. On the other hand, 5 mm·s^−1^ is the lowest possible value at which the irregularities of the cut surface will be investigated. The basic assumption at this speed is a significant increase in the parameters of the primary profile. The optimal wood cutting value observed by previous experiments is 10 mm·s^−1^.

**Figure 1:**
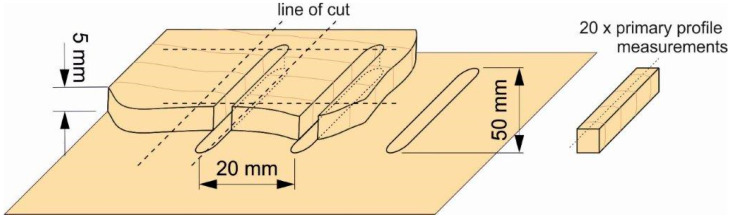
The area of measuring the surface quality of the kerfs cut by laser (left—method of cutting kerfs with a CO_2_ laser with subsequent cutting lines, right—the resulting sample for measuring the surface irregularities).

The focus point of the beam was placed in the middle of the thickness of the material. In Figure 1, it is possible to see a specific way of cutting the diagram used for the purpose of this paper. In the first step, kerfs in the direction perpendicular to the grain were cut into the samples with dimensions of 5 × 70 × 500 mm (thickness × width × length). In the second step, the created kerfs were cut perpendicularly as indicated by the dashed lines in the diagram (line of cut). The spacing of the kerfs of 20 mm was chosen so that the resulting samples for measuring the surface quality had a thickness of at least 5 mm and did not break during manipulation during the measurement. The length of the samples of 50 mm was chosen as a reference length for the observation of individual annual rings. The appearance of the resulting sample is shown in Figure 1 on the right. The resulting cross-section of individual types of wood was used to assess the quality. A total of 162 cross-section samples were obtained from the three types of wood, i.e., 54 for each type of wood.

### 2.2. Measurement of the Primary Profile

**Figure 2:**
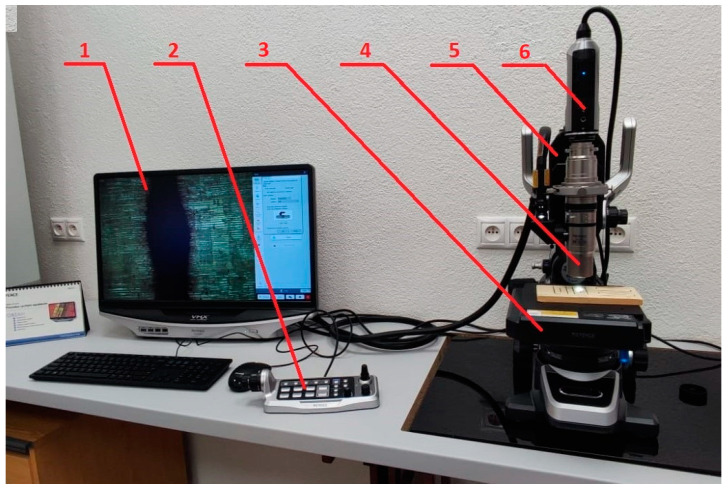
Construction of the Keyence VHX–7000 digital microscope (1—main unit; 2—console; 3—XYθ eucentric motorized stage; 4—wide-range zoom lens; 5—free-angle observation stand; 6—camera).

The Keyence VHX–7000 digital microscope (Osaka, Japan) was used as an optical measurement method to measure the primary cross-sectional profile. The construction of the measuring device can be seen in Figure 2.

Individual samples of the cross-section were scanned, and the resulting images of 5 × 18 mm in size were subsequently subjected to the optical measurement of the primary profile. The measurement of the primary profile was carried out in accordance with the technical standard EN ISO 21920 [32]. The interaction of the feed speed and the power of the laser on the surface of the oak, beech, and spruce wood was expressed through the P-parameters of the primary profile, namely Pa (arithmetic mean height), Pp (mean peak height), Pv (mean pit depth), Pz (maximum height), and Psm (mean profile element spacing). The primary profile contains asperities with two types of wavelengths: longer wavelength waviness and shorter wavelength roughness. Using the primary profile, it is thus possible to simultaneously evaluate the roughness of the cross-section resulting from cross-cut cellular elements as well as the anatomical waviness created between more charred earlywood wood and less charred latewood. Pa, like Ra, represents the arithmetic mean of the absolute values of the ordinates—that is, the height of the primary profile. However, the parameter itself does not provide any more detailed information about the heights of individual surface irregularities, but only describes the average value common to the entire profile. Thus, several profiles can have the same value of Pa despite significantly different irregularities. To evaluate the quality of the cross-section, the parameter Pz was also added, which describes the total height of the irregularities and is expressed as the sum of the height of the profile protrusion and the depth of the profile depression. In the case of a cross-section, this parameter is suitable for describing the height difference between earlywood (laser more damaged area) and latewood (laser melted, not damaged). The Pp and Pv parameters serve for a closer analysis of the Pz parameter, while Pp can be defined mainly by melted raised protrusions (hills) in latewood and Pv as dales primarily caused by the depth of individual cellular elements.

According to the standard EN ISO 21920 [32], the primary profile is defined as a profile derived from a profile trace, while a low-pass S-filter is used. This filter removes very small irregularities from the profile such as high frequency and low amplitude and wavelength components. The S-filter value (λs) was chosen to be 8 μm. The longer wavelength associated with waviness and the shorter wavelength associated with roughness were separated using a double Gaussian filter. The evaluation measurement length was 12.5 mm. Since the individual cross-section samples were described by average parameter values from several measurements, 20 profile traces in the radial direction (20 primary profiles) were measured on one sample. A total of 3240 values were measured for each investigated P-parameter.

### 2.3. Cross-Section Microscopic Analysis

For the purposes of microscopic analysis, a Keyence VHX-7000 digital microscope with a VHX-Z100R objective was used, zooming from 100× to 1000×. For image analysis, smaller sections from the cross-section were selected from the selected samples (the size of the section is visible within the 3D scale in the images in the results). Individual sections were created using the function of combining the image—so-called stitching. This function is automatically performed by the microscope, according to preset parameters and using an eucentric XYθ stage with motorized X and Y axes. The result is a larger area suitable for closer image analysis. The chosen zooms of the lens in the microscopic analysis were: 1000×—a detailed scan of the cut cellular elements with a focus on the melted parts burned by the laser; 500×—a scan of a larger area of the cross-section with a focus on the different melting in the zone of earlywood and latewood; and 200×—a scan of the cross-section focusing on height differences between more laser damaged earlywood and less damaged latewood.

As part of the second microscopic analysis, the images of the examined wood species were compared at a magnification of 1000×. The analysis was focused on a mutual image comparison of the height of the surface irregularities, which were created by the cellular elements present on the cross-section. The analysis aims to justify the magnitude of the values of individual measured P-parameters.

## 3. Results and Discussion

### 3.1. Measurement of the Primary Profile

**Figure 3:**
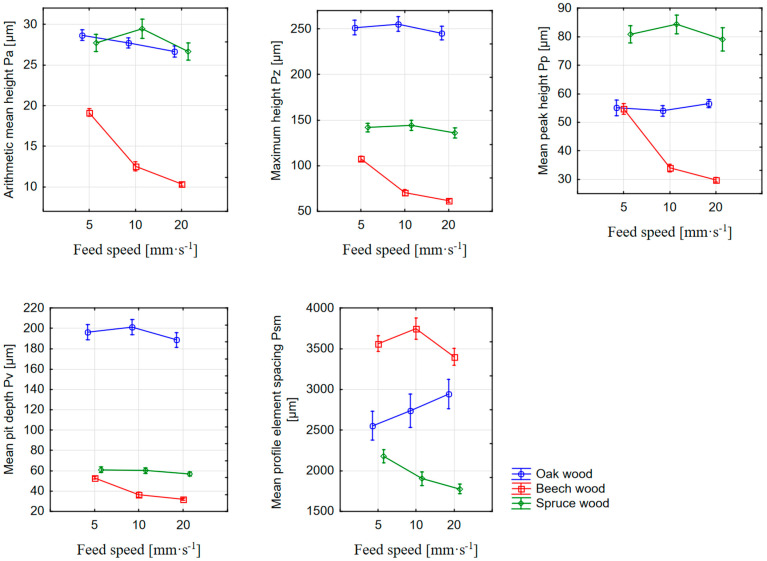
Dependence of the measured P–parameters on the feed speed of the laser and the type of wood. Vertical bars denote a 95% confidence interval for the mean.

**Figure 4:**
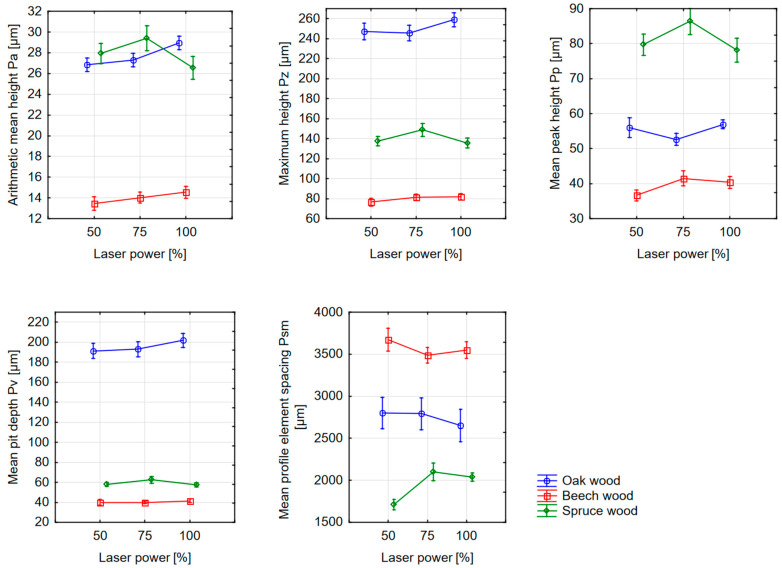
Dependence of the measured P–parameters on laser power and type of wood. Vertical bars denote a 95% confidence interval for the mean.

Before a statistical evaluation in the STATISTICA 12.0 software (Tulsa, OK, USA), the outliers for individual laser powers and feed speeds were removed from the dataset of measurements of P-parameters—Pa, Pv, Pp, Pz, and Psm, and missing data were measured. In the first step, the input data matrix was evaluated using methods of descriptive statistics. The results of the descriptive statistics in the form of arithmetic averages and 95% confidence intervals of P-parameters can also be seen in Figure 3 and Figure 4.

Subsequently, a two-factor analysis of variance with interaction (ANOVA) was performed. The investigated factors were the feed speed and the laser power, which affect the size of the P-parameter values. These two factors affect the quality of the surface created by the laser. When using ANOVA, it is necessary to fulfill conditions of normality, equality of variance, and the independence of measurements. The normality of the distribution of random variable values for all groups (combinations of factors affecting the P-parameter) was tested using the Shapiro–Wilk test. The test results showed a Gaussian distribution of values. Equality of variance at individual factor levels as the second condition of ANOVA was tested using Levene’s test. As a result of the test, the null hypothesis about the equality of variances was not confirmed, which was probably caused by the cross-section irregularities of different sizes in the individual measured profile traces. ANOVA is a robust technique, meaning that assumptions can be violated to some extent, but it can still be applied. The most important assumption for the use of ANOVA is the Independence of the values of the measured quantity, IIch in our case, is sufficient to be evaluated by a logical assessment. The results of the analysis of variance with interaction can be seen in Table 1. Based on these results, it can be concluded that all investigated factors—a type of wood, power, and feed speed—have a significant effect oI the investigated P-parameter in an interaction (*p* = 0.000 except for the parameter Pv, where in the interaction between the type of wood and power, *p* = 0.033). The effect of one factor is conditioned by the effect of another.

The basic premise of this paper on the influence of the feed speed was the decrease in the surface irregularities with an increasing feed speed. At a lower feed speed, the same intensity of the laser beam remains on the surface for a longer time, which leads to the degradation of the wood under the influence of the beam. More charred parts then create characteristic irregularities on the surface.

If the quality of the surface created by the CO_2_ laser cutting is defined separately through the arithmetic mean of the Pa parameter values, based on Figure 3, it is possible to state that the surface of the beech wood is of the highest quality. At the same time, Pa decreases with an increasing feed speed for all three types of wood. However, the issue of surface irregularities, which in this case are defined by the primary profile, must be understood in a broader context. As the arithmetic mean of the absolute values of the ordinate values, i.e., the height of the irregularities (value of the amplitude of the irregularities), Pa can be influenced by very deep dales or high hills in the irregularities. For this reason, there may be equality or great similarity in the values of the parameter Pa between different profiles. Therefore, it is necessary to supplement the measurements with other parameters, especially the amplitude parameters, which describe the heights of the individual parts of the irregularities (dales and hills).

The phenomenon wherein Pa values are approximately the same, but the height of the irregularities is significantly different, can be directly observed in Figure 3 (difference in the irregularities between spruce and oak wood). The measurements show that the mean peak height (Pp) was measured with spruce wood. Since the primary profile is a “mix” of longer and shorter wavelength irregularities, the resulting Pp value will be the difference between the profile reference line and the less burned latewood as well as unburned protruding fibers (fuzziness of the cross-section surface). Since the biggest differences between the density of earlywood and latewood are in spruce wood, depending on the parameters of the laser cutting, the biggest peaks will also appear. The sum of these heights with the lower values of the mean pit depth of the dales is also the reason why the spruce wood surface has the second highest measured maximum height (Pz). In most cases (except for oak wood), the size of Pp decreased with an increasing feed speed.

With the Pv parameter, it is possible from Figure 3 to observe a decrease in values with an increase in the feed speed. The parameter is related to the burnt earlywood (i.e., dale under the reference line of the primary profile) and to the depth of the cell elements on the cross-section. Higher values of the parameter at a lower feed speed can be justified by the longer-lasting intensity of laser radiation per unit area. Longer interaction between the beam and the wood structure caused a greater chipping of elements at the microscopic level and vice versa. The lowest Pv values for beech wood can be justified by the higher homogeneity between the nature of the earlywood and latewood compared to the spruce wood. However, the values measured for oak wood are significantly deviated. Since properties like those of beech wood can be expected, the sudden increase in values cannot be explained by the nature of the wood’s anatomy. To determine the causes of this phenomenon, it is necessary to subject the samples to a microscopic analysis of the surface.

The parameter Pz, defined as the sum of Pp and Pv, will be mainly related to irregularities between more burned earlywood and less burned latewood. From the graph below (Figure 3), it can be observed that, at a higher feed speed, the earlywood did not have time to degrade due to the influence of the laser beam, which resulted in a decrease in the maximum height of the irregularities. The higher Pz values of spruce wood compared to beech wood are thus logically justified. The deviation of the values in the case of oak wood is in this case caused by the high Pv values, which in the sum of the values also caused an increase in Pz.

In addition to the amplitude parameters of the primary profile, Psm was also evaluated as a parameter based on the profile element. From the point of view of surface microscopy, it is mainly related to the width of the burnt part, which correlates with the density distribution within the individual annual rings. The size of the Psm will thus also be related to the width of the annual ring. In the case of spruce wood, its width is from 0.5 mm to 2 mm [33]. For oak and beech wood, the width of the annual ring is from 2 mm to 5 mm. Again, Psm values were also measured in this range. From this point of view, the highest Psm value was measured for beech wood, whose annual rings had the largest width. At the same time, as the feed speed increases, the Psm values decrease for beech and spruce wood. From the measured values, however, it is not possible to determine the reason why the Psm values for oak wood increase with increasing feed speed.

From Table 1, it follows that the factors relating to the type of wood and laser power are statistically significant and in mutual interaction. The increasing power of the laser causes a greater amount of energy delivered in the form of radiation to the surface of the wood. This causes the greater burning of individual particles associated with the formation of surface irregularities. When assessing the primary profile by the Pa parameter, it is possible to claim, based on Figure 4, that with high-density wood, the increasing power also caused an increase in the Pa parameter. In addition to the difference between earlywood and latewood, this phenomenon can also be explained by the formation of burned-up melted parts, especially in the zone of denser latewood, which, as a form of irregularities, also increase the Pa parameter. The opposite situation occurred with spruce wood, where increasing laser power caused a decrease in Pa values. A possible explanation is that the higher output, and therefore the higher energy delivered to the cutting process, was able to increase the burning of the latewood, thus equalizing the difference between the earlywood and latewood zones.

The theory of the different effects of the laser beam on the zone of earlywood and latewood is also confirmed by the development of the values of the parameter Pz. They copy the development trend of the Pa parameter by the rate of increase and decrease. In this case, they also confirm the fact that, with approximately the same value of Pa, a different maximum height (Pz) can occur on the evaluated profile. From the graph below (Figure 4), it can be concluded that the smallest irregularities were on the surface of beech wood, which from this point of view, can be considered the highest quality of the surface cut by the CO_2_ laser. For a more detailed explanation of the Pz parameter, it is therefore important to examine the Pp and Pv parameters, i.e., the partial parts of the maximum height of the measured irregularities. Similarly, to the quantification of the influence of the feed speed, when a different power is applied to the surface of beech, spruce, and oak wood, the dose of energy in the form of a laser beam will have a different effect on the areas of earlywood and latewood.

In the case of the Pv parameter, there are no significant changes in beech and spruce wood due to the change in laser power. The spruce wood showed a higher Pv value compared to the more homogeneous beech wood, which again may be associated with earlywood, which appears as a dale below the reference line when evaluating the profile (the basis for the Pv parameter). As in the case of the influence of the feed speed on the P-parameter, the Pv values of the oak wood are the most deviated in this case. A sharp increase in values therefore needs to be subjected to a deeper microscopic analysis. As a result of the increase in the Pv parameter, the Pz values for oak wood also increased.

For the parameter Pp, different situations occurred in the three investigated types of wood. In the case of spruce wood, the protrusions (peaks) of the profile decreased with increasing laser power. A higher dose of energy delivered by the beam therefore smoothed the surface of the cross-section more. In the case of oak wood, there was a decrease in values at a laser power of 75%. Subsequently, however, with increasing power, the Pp parameter increased again, to a level of inequality slightly higher than at a power of 50%. In the case of beech wood, the Pp parameter showed an increase in profile peaks with increasing power. The phenomenon was probably caused by the greater burning of wood particles and approximately copies the development trend of the Pa parameter.

With an increasing laser power, there was also a change in the values of the Psm parameter. The highest values were measured for beech wood, where the width of the profile element again copies the annual growth of the wood and thus also the density distribution. Conversely, lower Psm values were measured for oak and spruce wood. In the case of beech and oak wood, the values of the Psm parameter decrease with increasing laser power. In the case of spruce wood, the individual annual rings had the smallest width, and therefore the alternation of peaks (latewood) and pits (earlywood) was more frequent. In this case, Psm values increased with increasing laser power.

**Figure 5:**
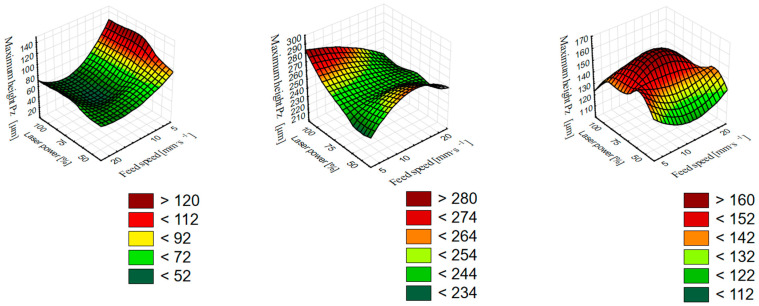
Three—dimensional surface plots of the combined influence of the feed speed and laser power on the total height of the irregularities Pz (from left to the right—beech, oak, and spruce wood).

In Figure 3 and Figure 4, the effects of wood type and laser cutting parameters on primary profile parameters were analyzed using two-factor ANOVA. From Figure 5, it is possible to see the interaction between both investigated factors—the feed speed and laser power on the Pz parameter (the interaction between these factors is statistically significant, with *p* = 0.001). Pz was chosen for this model as an indicator of the total height of the irregularities between more burned earlywood and less burned latewood. Pz as a parameter thus includes both profile dales (cellular elements) and profile hills (melts on the surface). The 3D surface plots show the places with critical Pz values (red color) as well as the places where the height of irregularities is the smallest (dark green color). From the graphs, it is possible to characterize such a choice of laser power parameters and feed speed, where surface irregularities will be the smallest and vice versa. For beech wood, the graph shows the most optimal setting of 75% laser power and a feed speed between 10 and 20 mm·s^−1^. For oak wood, it is a combination of 50% laser power and a feed speed of 5 mm·s^−1^ or 100% laser power and 20 mm·s^−1^. In the case of spruce wood, the lowest irregularities are at a combination of 50% laser power and a feed speed of 10 mm·s^−1^. The chosen type of graph proves to be essential for research into the optimization of cutting parameters and for the clear identification of places with low or, on the contrary, critical surface irregularities. The interpretation of P-parameter values under the influence of individual factors was described in the previous sections.

### 3.2. Microscopic Analysis of the Cross-Section Surface after CO_2_ Laser Cutting

**Figure 6:**
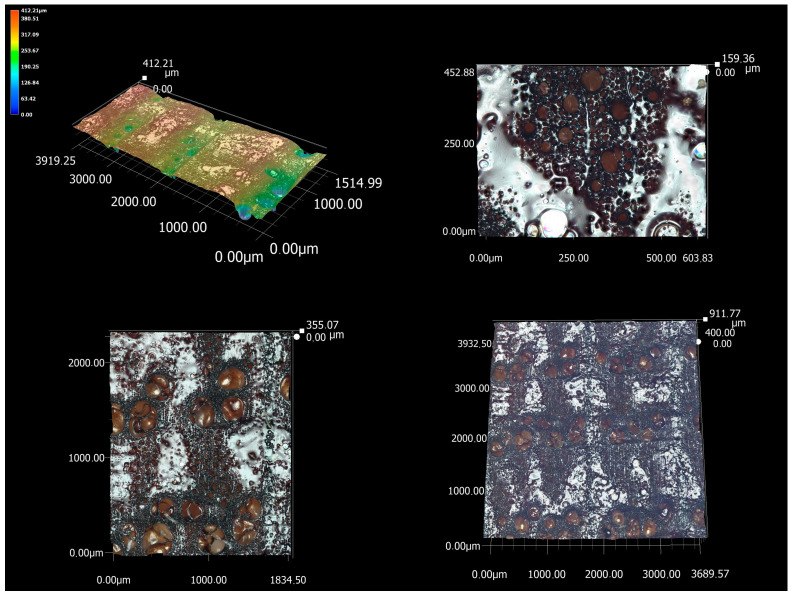
Microscopic analysis of a cross-section of oak wood (magnification of images clockwise: 200×, 500×, 200×, 1000×).

The results of the microscopic analysis of the cross-section are topographic images of the surface. They provide a deeper analysis of the dominant irregularities in this section as well as a mutual comparison of the cross-section surface of the three investigated wood species. At the same time, the analysis contains images with a very large magnification, on which it is possible to observe the interaction between the laser beam and the cellular elements present in this section. From Figure 6, it is possible to observe a cross-section of oak wood. The dominating cellular elements are mainly very wide earlywood vessels, whose diameter could be reflected in the increase in Psm values. From the analysis, it is possible to observe a clearly separated zone of less burned latewood (wood with a higher density) and more burned earlywood. These periodically alternating peaks and dales were reflected in all amplitude parameters in the irregularity’s measurement.

At a 200× magnification (bottom left corner of Figure 6), it is possible to observe clearly visible bands of laser-melted wood, which are mainly found in denser latewood. In the area of less dense earlywood, with unchanged cutting parameters, melted parts are only found in some places. The wood melt on the surface of the cross-section is the melted chemical components of wood, especially lignin. Lignin is primarily contained in the intercellular layer (middle lamella), which connects individual cells in wood. It is a thermoplastic substance that softens because of the very high temperatures of the laser beam on the machined surface. The laser beam itself can reach a temperature of over 650 °C when in contact with the surface of the wood [31]. Under certain conditions, the molten mass could cover the cellular elements and partially smooth the surface. However, the topographic image of the surface in this case showed that the melts appear as microscopic peaks, which increase the irregularities of the evaluated surface. In the process of removing wood at the point of contact with the laser beam, the least stable chemical component—hemicelluloses and cellulose—is also degraded. The high temperature of the laser beam acting on these chemical components causes structural changes in polysaccharides, mainly consisting of the advanced deacetylation and degradation of hemicelluloses. The FTIR analysis of laser-cut surfaces clearly demonstrated the presence of processes such as hemicellulose degradation, possibly demethoxylation and advanced condensation in the lignin structure [29]. The FTIR analysis of the laser-cut surface shows a lower content of polysaccharides and an increased content of lignin [29,30]. On the surface of the melt, it is also possible to observe circular holes through microscopic analysis. Most likely, these are places where carbon monoxide gases or water have leaked. These are immediately released from the cut site by the sublimation of the chemical components in the wood as a result of the high temperature achieved by the absorption of the laser beam energy [31]. These hot gases also damage the surface of the wood [34]. From the microscopic analysis, it is also possible to observe the significant charring of the laser-treated surface.

**Figure 7:**
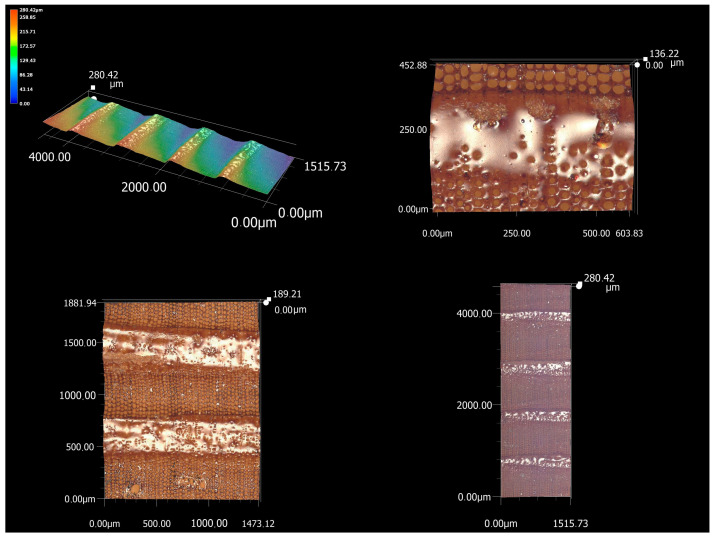
Microscopic analysis of a cross-section of spruce wood (magnification of images clockwise: 200×, 500×, 200×, 1000×).

Microscopic analysis of spruce wood showed very significant height differences in the cross-section (Figure 7). When investigating the influence of the feed speed and laser power, these became the cause of high values of the parameters Pp, Pv, and thus also Pz. From the analysis of the shape of the primary profile as well as the topographic image of the microscopic analysis itself, it is possible to see the gradual burn-off of the material in earlywood and the subsequent height jump in latewood, the burn-off of which is not so pronounced. This phenomenon is caused by the different densities of earlywood and latewood [35]. The density of earlywood is on average around 390 kg·m^−3^ according to [36] and 240 kg·m^−3^ according to [33], while the density of latewood is 640 kg·m^−3^ according to [36] and 604 kg·m^−3^ according to [33]. In both cases, latewood has a significantly higher density. The structure of spruce wood results in a gradual thinning of tracheids lumens towards latewood and a gradual increase in the thickness of the cell wall. With a thicker cell wall, its individual parts also grow, i.e., those with a higher proportion of lignin—the primary wall. During thermal degradation by laser beam, lignin softens and melts, which ultimately leads to the “flooding” of the surface with a honey-like consistency. Less burnt latewood is thus made up of densely stacked thick-walled tracheids, the burning of which requires a higher amount of energy.

With a constant dose of energy supplied to the volume of material in the process of creating a kerf, the significantly different density of wood within the annual ring is also manifested through significantly different values of thermophysical properties, which are strongly dependent on the density of the wood [37,38,39]. In this case, the difference in the thermophysical properties is responsible for the intensity of the heat removal process from the cut site. In the case of earlywood with low density, the significantly lower value of thermal conductivity is responsible for slow heat dissipation and its accumulation at the cut point. The surrounding structures around the place of formation of the cutting kerf act as insulation, as a result of which there is a significant increase in temperature and the thermal degradation of the material. The result is the removal of a larger volume of material, and an increase in the irregularities of the surface. In the case of latewood with a significantly higher density, the thermal conductivity also increases, as a result of which the heat created by the interaction of the laser beam with the material is significantly better dissipated to the more distant structures of the material and there is no increase in temperature. The result is a smaller volume of degraded material in latewood. Due to significant differences in the density of spruce wood, this process is one of the causes of large differences in the surface topography of the cutting surface, or surface quality indicators.

In the case of spruce wood, the effect of the laser on earlywood and latewood is differentiated, as represented by a sharp visible border. On one side, little carbonized earlywood is present, without the occurrence of melts, with dark cross-cut coils. On the other hand, latewood, unlike oak latewood, is melted almost over the entire surface and creates shiny strips of melt. These were also captured during microscopic analyses of other coniferous wood species [29]. Such a melt will likely prevent the further carbonization of the surface, and for the future degradation of not only the layer of melt, but also the layer of wood “below” it, it will be necessary to supply more energy and increase the heat (the melt will most likely be mostly made up of more heat-resistant lignin than polysaccharides). This can be achieved with a higher power of the laser beam, which confirms the measured fact that with a higher power of the laser, the Pp parameter will subsequently decrease—the melt and part of the latewood will burn off, thereby reducing its height and thus the height of the profile hills. Since the rate of decrease in Pv at higher power is not so significant, ultimately, the higher laser power caused a decrease in the maximum height of the irregularities—Pz.

**Figure 8:**
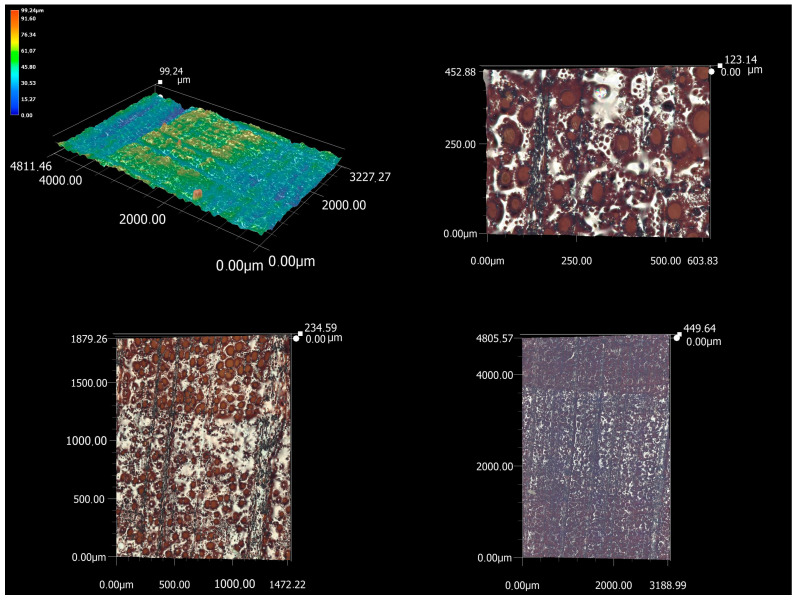
Microscopic analysis of a cross-section of beech wood (magnification of images clockwise: 200×, 500×, 200×, 1000×).

Beech wood subjected to microscopic analysis is characterized by higher homogeneity between the earlywood and latewood (Figure 8). There is not a sharply defined zone of less burnt latewood and more burnt earlywood. For this reason, the lowest values of the height of the irregularities were measured when measuring the Pz parameter. According to the measurements of the parameters of the primary profile, beech wood is thus the smoothest surface created by CO_2_ laser cutting. However, its surface is largely carbonized.

The rate of irregularities distribution will also be closely related to the rate of melt formation. Their distribution on the surface is irregular, scattered, and flows from the structure of beech wood as a characteristic diffuse-porous type of wood. From the detail of the microscopic analysis, the melts are located between the wide vessels, i.e., around thick-walled wood fibers. The degree of their melting is so high that, even in the case of microscopic analysis, it is impossible to capture them. In addition to the raised melts, the cross-section of beech wood also contains rays, which, compared to the surrounding cellular elements, form dales on the surface.

### 3.3. Microscopic Analysis of Cut Cell Elements

**Figure 9:**
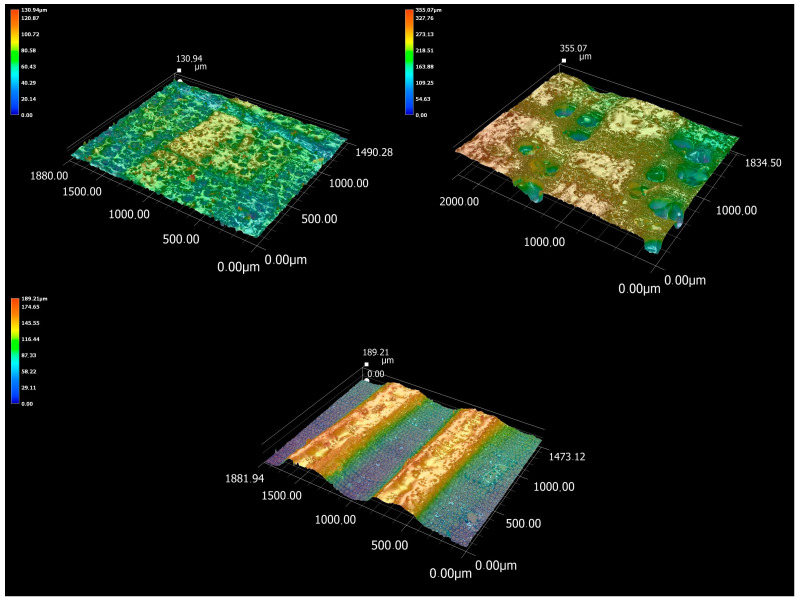
Microscopic analysis of beech (upper left corner), oak (upper right corner), and spruce wood (lower middle) at 1000× magnification.

After the measurement of P-parameters and microscopic analysis with topographical images of the CO_2_ laser-treated surface and the images of melts at a greater zoom, an additional microscopic analysis was performed focusing on the cut cell elements (Figure 9). The result of the analysis is a detailed representation of the cross-section surface cut by the laser. As can be seen from Figure 9, the height of irregularities was color-coded to scale. Dark blue areas are the lowest points on the scanned surface, as represented by the depth of the cross-cut elements. The red points are the highest points, as represented by the area of less burnt earlywood, specifically the frequent melts present. The analysis shows that the greatest height of irregularities is in oak, but is lower in spruce, and smallest in beech wood. With oak wood, it is possible from Figure 9 to observe the depth of individual vessels, which will significantly affect the amplitude P-parameters (Pp, Pv, and Pz). With spruce wood, the height difference between earlywood and latewood can be observed in particular. In the case of beech, the difference between earlywood and latewood is smaller, and the wood is slightly more homogeneous. The height of the irregularities on the topographic map is mainly caused by the fuzziness of the cut surface, i.e., fibers protruding from the laser-burned part.

However, with the investigated types of wood (especially beech and spruce wood), it is possible to observe that the actual depth of the transversely cut cell elements was not captured by the microscope. The reason is that the scanning range, the depth of field, as well as the working distance of the lens do not allow focusing on the “end” of the cut cellular elements. This error inherent in the measuring device was also caused by the fact that there is no surface inside the hollow cell elements from which the coaxial illumination of the microscope would be reflected. Without reflection, it is not possible to create an image with a CMOS sensor in a digital camera. The evaluated topographical surface thus appears to the device as “shallow”. The opposite case is oak wood with a high proportion of vessels with tyloses, from which the light was reflected back, which was reflected in the recording of the depth with a microscope. The mutual comparison of these types of wood thus becomes significantly distorted. A similar complication can also occur with stylus profilometers, when the tip of the measuring device “does not reach” the “end” of the cell elements and subsequently evaluates the values deviated from the actual state.

When evaluating the irregularities of the surface, it is necessary to use filters to define the wavelength by which we define the investigated irregularities. In this case, the cut cell elements represent an irregularity with a shorter wavelength, falling into the roughness region. On the contrary, the irregularities between more burnt earlywood and latewood have a longer wavelength, which means that, in the evaluation, they reach the area of waviness (anatomical waviness). Since the digital microscope did not capture cellular elements such as dales on the surface of beech and spruce wood, the imaged profile becomes “smoother” without short-wave components. This results in a significant distortion, especially of the amplitude P-parameters. In the case of oak wood partially, it represents a real surface; however, in the cases of beech and spruce wood, they cannot evaluate the hollows of cellular elements. The original assumption about the dependence between the short-wave components of the primary profile and the laser cutting parameters is thus limited to minor irregularities due to melts or the unburnt fuzziness of the surface, which, however, do not cause significant differences. The dominant irregularities of the cross-section thus become an anatomical waviness caused by more burnt earlywood and less burnt latewood. The latter falls with a longer wavelength into the area of the evaluation of waviness. It is caused by the different effects of laser radiation energy on earlywood and latewood, which is probably related to the difference in density of these two zones as well as to the different structures. One of the possible solutions to the research of cross-section irregularities after CO_2_ laser cutting is to focus on the evaluation of W-parameters, specifically the amplitude parameters of the waviness (maximum height of wave, arithmetic mean hIht, …). The mutual comparison of oak, beech, and spruce wood would not be distorted by differently recorded cellular elements. When measuring waviness, these would be attenuated by using an L-filter (λc), while form errors caused by laser cutting inaccuracies would be removed by an F-filter (λf).

However, the advantage of evaluating the irregularities with the primary profile turns out to be essential, with possible use especially on the radial or tangential section. The presented methodology is innovative because it can evaluate the complex effects of wood anatomy and laser cutting parameters on the surface, which is very important in woodworking. However, its certain negatives stem from the fact that, for the optimization of the cutting process, it is necessary to separately investigate the surface waviness component, which will become the subject of future research. Another advantage of the evaluation of the primary profile is the non-distortion of the surface profile by individual filters (λc, λf). The results thus reflect the real irregularities measured on the surface and lead to more accurate mutual comparisons.

The contribution of the presented paper was the definition of the quality of the surface (quantified through P-parameters) affected by the cutting conditions of the CO_2_ laser, the identification of the dominant surface irregularities, and the explanation of their causes in relation to the anatomical structure of the wood. The submitted paper mapped the origin of irregularities down to the microscopic level of wood. In this respect, our findings agree with the works by Kúdela et al. and Gurau et al. [29,30], who described the interaction between the laser and wood as a material through the microscopic structure (interaction with cell elements and cell walls). The work by Gurau et al. [29] also confirmed the decomposition of chemical substances at the submicroscopic level through FTIR analysis. This presented paper, in turn, expanded the mentioned issue with microscopic analysis and 3D topography of the surface. The analysis clearly showed the origin of the dominant cross-section irregularities. This paper also reached the same conclusions in the field of wood sublimation, burning, and formation of characteristic melts.

The presented paper is of benefit to the field of wood processing, as it explains the phenomena arising in laser cutting as a chipless method of wood cutting. The work explained in detail the statistical significance of the input parameters and their influence on the surface quality. At the same time, this article is a prerequisite for further research into cutting parameters and their modeling in relation to the quality of the created surface. The article investigated the action of the CO_2_ laser at lower feed speeds and lower powers, compared to the works of other authors [16,29,30]. From the results, it is possible to clearly determine which values of the feed speed and power parameters achieve the most optimal quality. The measured results confirm the claim that the quality deteriorates (the roughness parameters increase) with an increasing CO_2_ laser power, [30]. Rezaei et al. [16] also confirmed the improvement in the quality of the surface of beech wood with a moisture content of 8% with an increasing feed speed of the laser.

It follows from the works of the mentioned authors thaI surface irregularities are a feature strongly influenced by individual anatomical directions. This phenomenon results from anisotropy in relation to the different wood structures in the given directions. This greatly complicates predicting the effect of laser cutting parameters on the surface. A general question in the field of evaluating surface irregularities is the separation between anatomical irregularities related to the structure of the wood (and thus also anatomical directions) and processing irregularities, arising from the phenomena of the cutting process. Possible solutions result from Abbot’s curve [40] and the resulting parameters Rpk, Rk, and Rvk [29,41]. Rk is considered a parameter primarily related to the irregularities created by the cutting process, which is least related to the anatomical structure. Conversely, Rpk is related to surface fuzziness and Rvk mainly to deep pores. Evaluation through these parameters thus gives the closest answer to the question of what surface irregularities were caused by CO_2_ laser cutting. At the same time, the parameter Rk shows the highest values of the coefficient of determination and is thus suitable for predicting the effect of the laser on the surface. In the presented paper, these parameters were not measured, since the Keyence digital microscope as a measuring device does not allow their evaluation. Solving the question of the effect of wood anisotropy on surface irregularities will also strengthen prediction models. Yang et al. [18] also dealt with an advanced solution for predicting the effects of CO_2_ with a laser using a neural network. Another approach can be Gaussian random field modeling as a practical model [42,43]. However, each of the mentioned models first requires an initial set of measurements to determine the significance of the influence of individual parameters on surface irregularities.

## 4. Conclusions

In the presented paper, irregularities on the cross-section of beech, oak, and spruce wood after CO_2_ laser cutting were analyzed. The quality of the created surface was expressed through the parameters of the primary profile—Pa, Pv, Pp, Pz, and Psm. The monitored parameters of the laser were the laser power and the laser feed speed, both of which had a statistically significant effect on the P-parameter. At the same time, the ANOVA analysis revealed the statistical significance of the interaction between the laser power and the feed speed, which was expressed by 3D surface plots. The measured results confirmed the assumptions of other authors about the increase in surface roughness with the decreasing feed speed and the increasing laser power of the CO_2_ laser. Using 3D surface plots, it was possible to identify those combinations of the observed factors, in which the interaction of the factors resulted in a significant increase in the irregularities of the surface. These findings are also key to optimizing the quality of the surface created by laser cutting.

The proposed methodology for measuring the primary profIle proved its suitability for the analysis of the complex influence of laser cutting parameters. The primary profile was only slightly affected by the filters. By combining components with longer and shorter wavelengths, this methodology can assess the roughness and waviness of the created surface, which is very suitable for wood as a material. The P-parameters thus include all the irregularities created on the surface—including the irregularities resulting from the anatomical structure (different burning between earlywood and latewood), as well as the irregularities inherent in the laser cutting process (formation of melts). On the other hand, the disadvantage of this measurement method results from the error inherent in the measuring device. During the verification, it was found that the digital microscope could not optically capture the depth of the cellular elements in some types of wood, which resulted in a smoothing of the profile. This subsequently caused a significant decrease in some evaluated P-parameters. The scanned profile thus showed distortions in irregularities with a short wavelength (roughness), while it showed very good results in irregularities with a longer wavelength (waviness). With optical methods of measuring the irregularities of the cross-section, it is therefore more appropriate to focus on the waviness of the surface. The measurements include the dominant irregularities after laser cutting—the height differences between more charred earlywood and less charred latewood. However, the assessment of irregularities through the primary profile is very suitable for other cut surfaces, for example, radial or tangential surfaces, where it is possible to evaluate the surface from the anatomical-processing point of view of irregularities formation using this methodology.

The results of the microscopic analysis also confirmed the conclusions from the papers of other authors. Melts were identified on the laser-cut surface, the origins of which resulted from the complicated submicroscopic and microscopic structure of the wood. The range of melts, especially in latewood, was mainly related to the type of wood. In the case of beech wood, the melts were more scattered over the cut surface. In the case of spruce wood, they formed wide bands, while the latewood was almost completely melted on the surface, and in the case of oak wood, the melts were mainly present in the narrower vessels of the latewood. The benefit of this paper is also the use of 3D topographical analysis, which clearly proves the origins of individual surface irregularities. The analysis complements knowledge from stylus profilometers, where it is not possible to scan the surface microscopically, and the origin of the surface irregularities is thus very difficult to prove. That is why the optical methods of surface irregularity measurements using digital microscopes are very useful for the researching the quality of the created surface.

## Figures and Tables

**Table 1:** Two-factor analysis of variance (ANOVA).

Effect	Pa *p*-Level	Pp *p*-Level	Pv *p*-Level	Pz *p*-Level	Psm *p*-Level
Type of wood	0.000 **	0.000 **	0.000 **	0.000 **	0.000 **
Laser power	0.028 **	0.025 **	0.119 *	0.057 *	0.492 *
Feed speed	0.000 **	0.000 **	0.000 **	0.000 **	0.254 *
Type of wood × laser power	0.000 **	0.000 **	0.033 **	0.000 **	0.000 **
Type of wood × feed speed	0.000 **	0.000 **	0.000 **	0.000 **	0.000 **

* Statistically non-significant effect, ** Statistically significant effect.

## Data Availability

The data presented in this study are available upon request from the corresponding author.
